# Development and pilot evaluation of a structured curriculum for surgical handover

**DOI:** 10.1186/s12909-025-08044-3

**Published:** 2025-10-22

**Authors:** Jessica M. Ryan, Walter Eppich, Dara O. Kavanagh, Anastasija Simiceva, Tom V. McIntyre MRCSI, Deborah A. McNamara

**Affiliations:** 1 RCSI University of Medicine and Health Sciences, St. Stephen’s Green, Dublin, Ireland; 2 RCSI Department of Surgical Affairs, St. Stephen’s Green, Dublin, Ireland; 3 The Bon Secours Hospital, Glasnevin Hill, Glasnevin, Dublin, Ireland; 4 Faculty of Medicine, Dentistry and Health Sciences, University of Melbourne, Melbourne, Australia; 5 Department of Surgery, Tallaght University Hospital, Tallaght, Dublin, Ireland; 6 Department of Surgery, Beaumont Hospital, Beaumont, Dublin, Ireland

**Keywords:** Surgical education, Handover, Handoff, Surgical handover, Medical education

## Abstract

**Background:**

Effective handover communication is a core professional competency in graduate medical education, yet very few junior doctors working in surgery receive formal training. A structured curriculum was developed and piloted to teach best practices in surgical handover, based on a recognised curricular framework.

**Methods:**

The study was carried out at two academic tertiary hospitals in Dublin, Ireland. Interns attending mandatory weekly teaching sessions participated in a 60-minute intervention combining didactic teaching, video demonstration, small group simulation, and facilitated discussion. Self-reported confidence in delivering and participating in handover was assessed using pre- and post-session surveys. Post-session feedback on curriculum content and format was also collected.

**Results:**

A total of 59 interns attended the teaching sessions, with 35 providing paired pre- and post-session data. Self-reported confidence significantly improved across all assessed domains assessed (p<0.001), including confidence in handing over to peers and senior colleagues, asking clarifying questions during handover, and providing a summary or ‘readback’ at the end of handover. Feedback from 46 participants indicated that the session was well-received, with video demonstrations and simulated practice rated most helpful. Didactic teaching and peer feedback were rated least helpful. A majority (76.1%; n=35) reported that the session would lead to changes in their handover practice.

**Conclusions:**

This pilot study showed that a simulation-based curriculum is effective in improving interns’ self-reported confidence in delivering and receiving surgical handover. The teaching session was well-received, easily integrated into existing institutional infrastructure, and required minimal resources to carry out.

**Supplementary Information:**

The online version contains supplementary material available at 10.1186/s12909-025-08044-3.

## Introduction

Clinical handover is a recognised priority for patient safety [1]and has serious implications if not performed well [2–4]. Effective handover communication is a core professional competency that should be taught and assessed during graduate medical education [5]. Surgical handover involves the transfer of patient information and responsibility between clinicians [6] who work in surgical specialties. It presents unique challenges, including tight time constraints, competing clinical demands at the time of handover[7], a high level of patient acuity, and frequent transitions of care throughout the perioperative period.

Active involvement of early career doctors in handover has clear benefits for patient care [8]. Yet, despite its importance, only 11% of junior doctors working in surgery receive formal handover training [7], and until recently, no evidence-based guidance was available to support the development of dedicated curricula. A recent review by the authors proposed recommendations regarding the optimal approach, format, and content for educational programmes in surgical handover [9]; however, these have yet to be evaluated in practice.

To address this gap, a structured curriculum was developed and piloted based on these recommendations, designed to teach best practices for surgical handover. This study aimed to pilot this curriculum during routine in-hospital intern teaching and to assess its impact on participants’ self-reported level of confidence in delivering and participating in surgical handover. Findings from this study will inform further refinement of the curriculum and implementation on a larger scale.

## Methods

A curriculum for teaching best practices in surgical handover practices was developed using a recognised framework [9] and piloted at two hospital sites using a quasi-experimental pre-post study without a control group. The teaching sessions were carried out on the 23^rd^ of January 2024 (Site A) and the 8^th^ of February 2024 (Site B). Prospective study approval was received at both sites (CA2023/136 and 3714) and the GREET guideline for reporting evidence-based practice educational interventions and teaching [10] was followed to guide reporting of this study. 

### Setting and population

This study was conducted at two academic tertiary referral centres in Dublin, Ireland, with 500 and 820 beds, and catchment populations of 644,000 and 290,000 people, respectively.

Both sites carry out weekly, one-hour, lunchtime intern teaching sessions for which attendance is mandatory. The curriculum was delivered during two of these sessions, one in each hospital site, in January and February 2024. The teaching topic was pre-approved for inclusion by intern teaching coordinators from the Royal College of Surgeons in Ireland and Trinity College Dublin (one coordinator at each site). Participants included interns working across all hospital departments and specialties who attended the in-person teaching sessions. Food was provided to interns as the teaching sessions are scheduled to happen during lunch.

### Resource use

The teaching session was delivered using existing institutional infrastructure. It involved a one-hour session facilitated by two staff members (a primary and an assistant instructor), with approximately 60 min of preparation time in advance. Supporting materials included printed handover documents and pre-recorded video demonstrations, which required one hour of filming. Standard audiovisual equipment was used (projector with PowerPoint slides, ©Microsoft 2025), and interactive polling was conducted using free online software. Sessions were held in lecture theatres with internet access, booked for a one-hour duration.

### Curriculum description

The curriculum was developed using a recognised framework [9] and was grounded in social constructivism learning theory, which asserts that knowledge is built through social interactions and collaboration [11]. It was delivered by a primary instructor (JMR) who was completing a Doctor of Philosophy in surgical handover and had almost 10 years of experience working in surgery at the time. The assistant instructor (AS) was a research assistant on multiple surgical handover projects and has a Social Sciences background, with experience in education, policy, and practice relating to children, young people, and adults.

The curriculum was delivered over a 60-minute interactive teaching session designed to introduce best practices for surgical handover and combined didactic teaching, video demonstration, small-group simulation exercises, and facilitated group discussion (Table 1). Teaching began with a brief overview of the definition and importance of clinical handover, followed by the SIPS Surgical Handover System, a structured four-step approach designed to guide effective communication during handover meetings between incoming and outgoing staff. The steps include: (1) highlighting Sick patients at the start of the meeting; (2) using the ISBAR method (Identity, Situation, Background, Assessment, Recommendation) [12] to present each patient; (3) providing a Priority list of tasks and patients requiring surgical intervention; and (4) having a member of the incoming team briefly Summarise the key handover points [13]. Role-play videos created by the instructors, which demonstrated examples of suboptimal and effective handover, were then shown to stimulate discussion around common errors and effective strategies.

Interns then engaged in small group (n=3) simulated verbal handover practice using realistic examples of three patient cases included in sample handover documents. Each participant had an opportunity to deliver and receive a simulated handover, and provide peer feedback. The session concluded with group discussion and reflection on the simulation exercises, followed by repeat polling to capture changes in perception (Additional File 1.pdf) and feedback (Additional File 2.pdf) on the session.

**Table 1:** Surgical handover curriculum

Time	Activity
00:00–00:15	**Introduction and didactic teaching **Session introduction, interactive poll, and 10-minute slide-based lecture covering the principles and importance of handover, including steps of the SIPS Surgical Handover System [13]
00:15 − 00:25	**Video demonstration and group discussion** Video demonstration of poor and well-executed handovers, followed by a group discussion of video content
00:25 − 00:35	**Preparation for simulated practice **Participants divided into groups of three, where each person was provided with an example of a written handover document. The upcoming simulation exercise was explained, and they were given time to familiarise themselves with patient information. A visual aid for the handover method was made available (using slides and distributed cards for ID lanyards)
00:35 − 00:50	**Simulated handover practice and peer feedback** Each group conducted three simulated handovers (4 min each), with 1-minute for peer feedback and discussion after each
00:50 − 00:60	**Session debrief** A whole-group discussion was then carried out, followed by a repeat interactive poll, and collection of participant feedback on the session

Table 1 caption. This table provides a description of the curriculum with timings of each component

### Outcome measures and data collection

The primary outcome was interns' self-reported level of confidence in delivering and participating in surgical handover, which reflects a Kirkpatrick level I outcome [14]. These were assessed using pre- and post-session surveys (polls) with 10-point Likert scales. The survey items were developed by the author team based on prior research and experience in surgical handover [7, 8, 15], and aligned with international guidelines [1, 16]. They focused on key competencies such as providing a handover summary and posing clarifying questions during handover (Additional File 1.pdf).

The secondary outcome was participant feedback on the class content and format, and was assessed at the end of the session using an 8-item survey developed through a review of previous similar studies [17–19] (Additional File 2.pdf). Pre- and post-session surveys were distributed using QR codes and responses were recorded anonymously. Pre-session surveys additionally captured information on participants' current clinical posts (e.g., surgical, medical, other), prior handover education or training, and familiarity with the taught surgical handover method.

### Statistical methods

Data were analysed using Stata (17.0©2021, StataCorp, Texas). Categorical data were presented as absolute values and percentages, and continuous data as mean (standard deviation, SD). Comparative analyses were performed using chi-squared test for categorical data, while Likert scale ordinal data were coded numerically and analysed using paired t-tests. Parametric tests are considered more suitable and more robust in the assessment of Likert scale data, even when those data are not normally distributed [20, 21]. All tests of significance were two-tailed, with *p*<0.05 indicating statistical significance. Given the pilot nature of this study, no formal sample size calculation was performed.

## Results

### Participant characteristics

A total of 59 interns attended the teaching sessions (Site A: *n* = 27; Site B: *n* = 32), all of whom responded to at least one in-session survey question. Paired pre- and post-session polling data were available for 35 participants (59.3%). Demographic data were provided by 38 interns (64.4%); of these, 43.2% (*n* = 16) were working in surgical posts at the time of the session, 51.3% (*n* = 19) in medical specialties, and 5.4% (*n* = 2) in other departments. Only five interns (13.9% of 36 respondents to this question) reported receiving formal handover training previously.

### Self-reported confidence in handover

There was a statistically significant improvement in self-reported confidence across all four domains assessed (giving handover to another intern, giving handover to a senior colleague, asking clarifying questions during handover, and summarising to the team at the end of the handover meeting) following the teaching session (*p* < 0.001; Table 2). Within-subject effect sizes ranged from moderate (Cohen’s dz = 0.68 for posing clarifying questions) to large (dz = 1.14 for confidence in handing over to a senior colleague). Observed post-hoc power for each outcome was high (97%–100%), indicating that the sample sizes were sufficient to detect the observed changes at α = 0.05.

**Table 2:** Summary of handover confidence ratings pre- and post-teaching session

Question	*n*	Pre-teaching Mean (SD), CI Median (IQR)	Post-teaching Mean (SD), CI Median (IQR)	*P* ^a^	Cohen’s dz^b^	Post-hoc power (observed)^c^
How comfortable would you be giving patient handover to another intern?	31	8 (1.3), 7.5–8.5 8 (7–9)	9 (0.8), 8.7–9.3 9 (8–10)	< 0.0001	1.05	99%
How comfortable would you be giving patient handover to a senior colleague?	34	6.3 (1.5), 5.7–6.8 7 (5–7)	7.4 (1.5), 6.9–7.9 7.5 (7–8)	< 0.0001	1.14	100%
How comfortable would you be posing necessary clarifying questions during team handover?	35	6.4 (2.1), 5.6–7.1 7 (5–8)	7.5 (1.7), 6.9–8.1 8 (6–9)	0.0003	0.68	97%
How comfortable would you be providing a summary to your team at the end of a handover meeting?	27	6.5 (1.8), 5.8–7.2 7 (5–8)	7.5 (1.4), 6.9 – 8.0 7(6–9)	0.0004	0.79	97%

Table 2 caption. Table 2 provides a summary of intern handover confidence ratings pre- and post-teaching session (measured on a 10-point Likert scale)

*CI* Confidence interval, *SD* Standard deviation

^a^Analysed using paired t-test

^b^Cohen’s dz = mean of paired differences ÷ SD of differences; interpreted as small (0.2), medium (0.5), large (0.8)

^c^Post-hoc (observed) power = calculated from observed mean difference, SD of differences, and paired sample size for each outcome (α = 0.05, two-tailed)

### Attendee feedback

Post-session feedback was received from 46 interns (78%). Interns reported that the session was useful to clinical practice (mean Likert score: 4.2 ± 1.0), provided an effective review of the topic (4.1 ± 1.1), and would recommend it to others (3.9 ± 1.2).

In terms of class content, video examples of handover were rated the most helpful (4.1 ± 0.8), followed by simulated practice (3.8 ± 1.1). Didactic slides (3.6 ± 1.0) and peer feedback (3.4 ± 1.1) were rated lowest. A majority (76.1%; *n* = 35) reported that the session would lead to changes in their handover practice. Cronbach’s alpha for the 7-item Likert scale for attendee feedback (Additional File 2.pdf) was 0.9, indicating excellent internal consistency.

#### Open-text comments

Five interns (10.8%) provided open-text comments, highlighting the need for senior leadership to model improved handover behaviours:

“Change in culture should come from top down.”

– Intern B1.

One participant noted the session’s relevance may vary depending on clinical role:

“Applies better to regs and surgical interns. Not relevant to general interns.”

– Intern B2.

Another pointed out environmental and contextual barriers:

“Better not to hand over at ED nurses station [because it is] loud busy and you’re regularly interrupted”

– Intern B2.

Participants suggested improvements, including more time for simulated practice and more realistic and complex case materials:

“The simulated part was a little too rapid.”

– Intern B3

“3 patient handover wouldn't be the same as 7+ patients (which is the norm). Would like more tips for a longer and more complicated handover.”

– Intern B4.

One intern commented positively on the teaching approach:

“More tutorials should be in the same style.”

– Intern A4.

## Discussion

This pilot study showed that the previously described curricular framework for surgical handover [9] can effectively be used to develop a simulation-based teaching session for interns, leading to improvements in self-reported confidence (Kirkpatrick Level I outcome) in delivering and receiving surgical handover. Video demonstrations and simulated handover practice were rated highest in terms of content and methods used, with the provision of peer feedback rated the lowest. Interns reported high levels of satisfaction with the session and the majority felt that it would lead to changes in their practice. Survey responses suggest that simulated practice should be longer, using more complex scenarios, and that time spent on didactic teaching should be reduced.

Most participants had not received prior handover training, consistent with previous literature [7]. The findings reported here reinforce the need for structured, formal training in handover as part of graduate medical education [5] and support simulated handover practice in enhancing learner confidence, as well as the value of video demonstrations as a teaching tool [22]. In-person simulation, which is preferred by students [4], may be particularly appropriate for first-time learners [9].

This curriculum can be adapted for use in lower resource settings. It was easily incorporated into existing institutional teaching infrastructure and required minimal resources to carry out. In teaching settings without audiovisual equipment or internet access, in-person role-played handover demonstrations could be used instead of video. Given that students do not rate didactic teaching highly [4] and slide-based presentations may negatively impact learning [23], the introductory component of the session could also be conducted without slides to further reduce required resources. These factors make the curriculum particularly accessible for resource-limited settings.

Student feedback suggests that modifications to the curriculum are required, particularly in the format of simulation, which was perhaps too simplistic, and may not have given students enough time to familiarise themselves with patient details, deliver an effective handover, and receive feedback. The feasibility of expanding simulations within the one-hour teaching block may be limited; however, reducing didactic content could free time for more complex scenarios in future studies. Interestingly, although previous work suggests that peer feedback is effective [9], the current findings indicate otherwise, suggesting that while feedback is well-received, it may be better delivered on an individual level by session faculty as in previous studies [22]. 

Limitations of this study include the incomplete survey data, with paired pre- and post-responses available for only 59.3% of participants. Missing responses may have been due to late arrivals to the session (thus missing real-time polling), which could introduce bias. Additional limitations include the reliance on self-reported (Kirkpatrick level I) outcomes rather than objective changes in behaviour or patient outcomes [14]. Potential bias may also have arisen from the provision of food during sessions; however, as teaching was scheduled during the interns’ lunch hour, food provision represented standard institutional practice. This study was also conducted in a single geographic area with a small number of learners, limiting generalisability. Although the curriculum focused on surgical handover, fewer than half of participants were in surgical posts. However, the handover method which was taught is broadly applicable and likely relevant to non-surgical interns as well. Future studies should assess higher level Kirkpatrick outcomes, including retention of teaching, real-world impact on handover quality and patient outcomes [14] using a revised curriculum informed by the above feedback. Finally, this study did not include a control group, which limits our ability to attribute observed changes in learner confidence solely to the intervention. Future evaluations should incorporate a comparison group to strengthen causal inferences regarding the impact of the intervention.

## Conclusion

This pilot study showed that a simulation-based curriculum is effective in improving interns’ self-reported confidence (Kirkpatrick level I outcome) in delivering and receiving surgical handover. The teaching session was well-received, easily integrated into existing institutional infrastructure, and required minimal resources to carry out. Participant feedback highlighted areas for improvement, particularly around simulated practice and the receipt of performance feedback. While promising, these findings should be interpreted cautiously given the study’s limitations. Future evaluations should incorporate higher-level Kirkpatrick outcomes, including objective behavioural changes and patient outcomes, and include a control group where possible. Importantly, the curriculum’s minimal resource requirements suggest strong potential for scalability across a variety of training settings.

## Supplementary Information

Supplementary Material 1. Additional File 1.pdf Copy ofpre- and post-session survey with 10-point Likert scales to assess interns' self-reported level of confidence in delivering and participating in surgical handover

Supplementary Material 2. Additional File 2.pdfCopy of post-session survey to assess participant feedback on the class content and format

## Data Availability

All data generated or analysed during this study are included in this published article and its additional files.
