# Supplementary material for: Development and pilot evaluation of a structured curriculum for surgical handover
Source: BMC Med Educ. 2025 Oct 22;25:1482. doi: 10.1186/s12909-025-08044-3 (PMC12542472; doi:10.1186/s12909-025-08044-3)
Supplement: Supplementary file 1 — Supplementary Material 1. Additional File 1.pdf Copy ofpre- and post-session survey with 10-point Likert scales to assess interns' self-reported level of confidence in delivering and participating in surgical handover [file 12909_2025_8044_MOESM1_ESM.pdf]

Intern teaching attendee feedback - Tallaght

1. Please indicate your agreement with each of the following statements

	Strongly disagree	Disageee	Neither agree nor disagree	Agree	Strongly agree
This class gave an effective review of handovers					
This class was useful to clinical practice					
I would recommend this class to others					

2. Please indicate how helpful you found each of the following class elements

	Not at all helpful	Slightly helpful	Somewhat helpful	Very helpful	Extremely helpful
Didactic teaching (slides)					
Video examples of good and bad handover with discussion					
Simulated handover practice in groups					
Peer feedback (receiving feedback on your performance from other interns)					

3. Will your handover practice/technique change after attending this class?

- Yes
- No
- I don't know

4. Is there anything you would change about this class?

5. Anything else you would like to add?
