# Supplementary material for: Development and pilot evaluation of a structured curriculum for surgical handover
Source: BMC Med Educ. 2025 Oct 22;25:1482. doi: 10.1186/s12909-025-08044-3 (PMC12542472; doi:10.1186/s12909-025-08044-3)
Supplement: Supplementary file 2 — Supplementary Material 2. Additional File 2.pdfCopy of post-session survey to assess participant feedback on the class content and format [file 12909_2025_8044_MOESM2_ESM.pdf]

Part one

Mentimeter

How comfortable would you be giving patient handover to another intern?

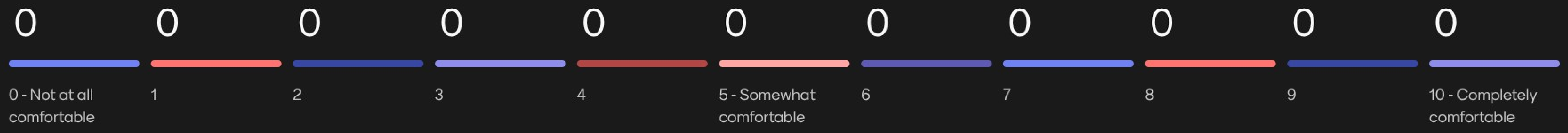

Mentimeter

How comfortable would you be giving patient handover to a senior colleague?

0 - Not at all comfortable	1	2	3	4	5 - Somewhat comfortable	6	7	8	9	10 - Completely comfortable
0	0	0	0	0	0	0	0	0	0	0

Mentimeter

How comfortable would you be posing necessary clarifying questions during team handover?

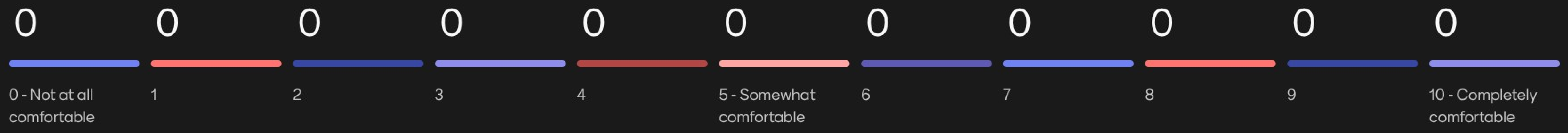

Mentimeter

How comfortable would you be providing a summary to your team at the end of a handover meeting?

0 - Not at all comfortable	1	2	3	4	5 - Somewhat comfortable	6	7	8	9	10 - Completely comfortable
0	0	0	0	0	0	0	0	0	0	0

Part two

How comfortable would you be giving patient handover to another intern?

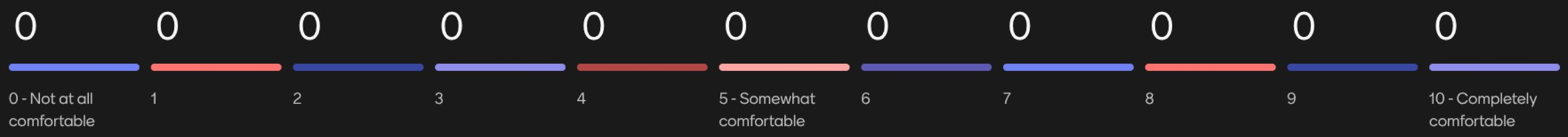

Mentimeter

How comfortable would you be giving patient handover to a senior colleague?

0 - Not at all comfortable	1	2	3	4	5 - Somewhat comfortable	6	7	8	9	10 - Completely comfortable
0	0	0	0	0	0	0	0	0	0	0

How comfortable would you be posing necessary clarifying questions during team handover?

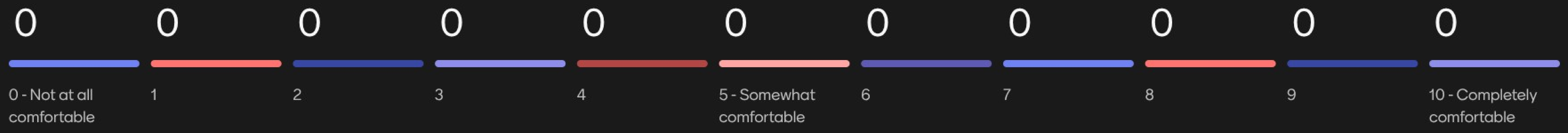

Mentimeter

How comfortable would you be providing a summary to your team at the end of a handover meeting?

0 - Not at all comfortable	1	2	3	4	5 - Somewhat comfortable	6	7	8	9	10 - Completely comfortable
0	0	0	0	0	0	0	0	0	0	0
